# Activation of Mechanistic Target of Rapamycin (mTOR) in Human Endothelial Cells Infected with Pathogenic Spotted Fever Group Rickettsiae

**DOI:** 10.3390/ijms21197179

**Published:** 2020-09-29

**Authors:** Abha Sahni, Hema P. Narra, Sanjeev K. Sahni

**Affiliations:** Department of Pathology, University of Texas Medical Branch (UTMB), Galveston, TX 77555-0609, USA; hpnarra@utmb.edu

**Keywords:** Akt (protein kinase B), endothelial cells, mTOR, protein kinase C, Rickettsia

## Abstract

Attributed to the tropism for host microvascular endothelium lining the blood vessels, vascular inflammation and dysfunction represent salient features of rickettsial pathogenesis, yet the details of fundamentally important pathogen interactions with host endothelial cells (ECs) as the primary targets of infection remain poorly appreciated. Mechanistic target of rapamycin (mTOR), a serine/threonine protein kinase of the phosphatidylinositol kinase-related kinase family, assembles into two functionally distinct complexes, namely mTORC1 (Raptor) and mTORC2 (Rictor), implicated in the determination of innate immune responses to intracellular pathogens via transcriptional regulation. In the present study, we investigated activation status of mTOR and its potential contributions to host EC responses during *Rickettsia rickettsii* and *R. conorii* infection. Protein lysates from infected ECs were analyzed for threonine 421/serine 424 phosphorylation of p70 S6 kinase (p70 S6K) and that of serine 2448 on mTOR itself as established markers of mTORC1 activation. For mTORC2, we assessed phosphorylation of protein kinase B (PKB or Akt) and protein kinase C (PKC), respectively, on serine 473 and serine 657. The results suggest increased phosphorylation of p70 S6K and mTOR during *Rickettsia* infection of ECs as early as 3 h and persisting for up to 24 h post-infection. The steady-state levels of phospho-Akt and phospho-PKC were also increased. Infection with pathogenic rickettsiae also resulted in the formation of microtubule-associated protein 1A/1B-light chain 3 (LC3-II) puncta and increased lipidation of LC3-II, a response significantly inhibited by introduction of siRNA targeting mTORC1 into ECs. These findings thus yield first evidence for the activation of both mTORC1 and mTORC2 during EC infection in vitro with *Rickettsia* species and suggest that early induction of autophagy in response to intracellular infection might be regulated by this important pathway known to function as a central integrator of cellular immunity and inflammation.

## 1. Introduction

Pathogenic *Rickettsia* species are classified as Gram-negative and obligate intracellular bacteria divided into four major subgroups, including spotted fever and typhus represented by causative agents of Rocky Mountain spotted fever (*R. rickettsii*), Mediterranean spotted fever (*R. conorii*), epidemic typhus (*R. prowazekii*), and endemic typhus (*R. typhi*). Natural transmission of rickettsial diseases to humans as accidental hosts primarily involves arthropod vectors, including ticks, lice, fleas, and mosquitoes [1,2,3]. Rickettsial infections are now known to occur across the globe, although resultant disease severity varies depending on the virulence of the pathogen. Following their introduction at the tick bite site(s), spotted fever group rickettsiae invade the cells of the dermis, from where they spread to blood vessels where they primarily target microvascular ECs lining the entire vascular tree within the host. Consequently, prominent pathophysiological characteristics of rickettsial diseases can best be summarized as generalized vasculitis, punctuated by increased expression of endothelial surface adhesion molecules resulting in the recruitment and extravasation of leukocytes to foci of infection, release of powerful vasoactive pro-coagulatory mediators and pro-inflammatory cytokines, compromised vascular permeability, and disseminated vascular inflammation leading to tissue edema and vital organ dysfunction [1]. A comprehensive analysis of pathogen interactions with microvascular endothelium as the preferred, primary host niche thus acquires tremendous importance in furthering our understanding of the pathogenesis of human rickettsioses.

The mammalian/mechanistic target of rapamycin (mTOR) is an essential serine/threonine kinase, which functions as a central regulator of cell metabolism, growth, proliferation, and initiation of translation [4]. As a member of the phosphatidylinositol (PI) kinase-related family, mTOR assembles into two functionally distinct complexes. In complex 1, mTOR associates with rapamycin-sensitive regulatory associated protein of mTOR (raptor) and mammalian lethal with sec18 protein 8 (mLST8), whereas mTORC2 is comprised of rapamycin-insensitive companion of mTOR (rictor), stress-activated protein kinase interacting protein 1 (SIN1) and mLST8 [4,5]. Functionally, mTOR signaling integrates both intracellular and extracellular signals to regulate autophagy and inflammatory responses [6]. 

Autophagy represents a fundamentally important eukaryotic homeostatic mechanism involved in the determination of inflammatory responses and innate as well as adaptive immunity [7]. In the setting of infection, autophagic cascade plays a context-specific role by either contributing to the elimination of intracellular microorganisms or potentiating their growth and replication by serving as a source of nutrients [8]. A seminal study focused on investigating the roles of mTOR in the regulation of innate inflammatory response has further suggested its involvement in the regulation of pro-inflammatory cytokines via nuclear transcription factor-kappa B (NF-κB) and an anti-inflammatory cytokine interleukin-10 (IL-10, also known as cytokine synthesis inhibitory factor) via signal transducer and activator of transcription 3 (STAT-3) [9]. In light of this important finding and our published work on the activation of both NF-κB and STAT-3 signaling pathways during infection of human ECs with *R. rickettsii* and *R. conorii* [10,11], this study was undertaken to investigate the activation of mTOR complexes C1 and C2 and induction of autophagy using established in vitro models of infection.

## 2. Results

**Figure 1:**
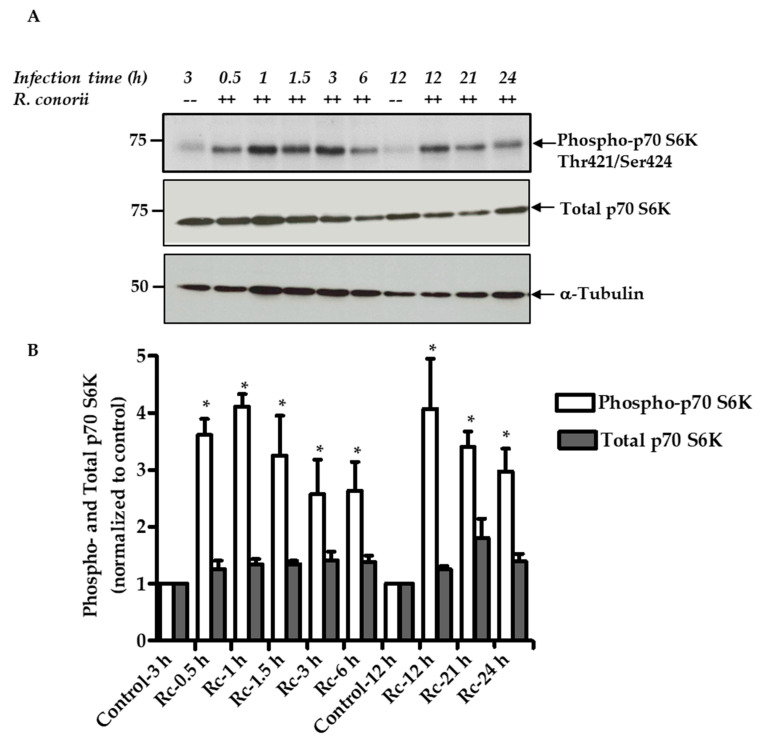
Mechanistic target of rapamycin complex 1 (mTORC1) activation in *R. conorii*-infected endothelial cells (ECs). (**A**). Confluent monolayers of human umbilical vein endothelial cells (HUVECs) were either mock infected (--) or infected with *R. conorii* (++) at a multiplicity of infection (MOI) of 5 plaque-forming units (PFUs) per cell for various times as indicated. Protein lysates were then prepared by cell lysis and subjected to immunoblot analysis with antibodies against phospho- and total p70 S6 kinase (p70 S6K). An α-tubulin antibody was used as a loading control to account for any variations in sample loading on different gel lanes. Relative positions of nearest molecular weight markers (kDa) from the protein ladder are displayed on the left flank of the gel. Results from a representative blot (*n* ≥ 3) are presented. (**B**). Quantitative band densitometric analysis of phospho- and total p70 S6 kinase during *R. conorii* (Rc) infection of host ECs is shown as a function of time. The values are presented as the mean ± standard error of the mean (SEM) for at least three independent experiments. For comparison, basal levels in mock-infected cells (controls) were assigned a value of 1. The asterisk (*) indicates statistically significant changes (*p* ≤ 0.05) in *R. conorii*-infected cells compared with the baseline in corresponding samples from uninfected ECs. Similar results were obtained with *R. rickettsii*.

The mTOR pathway plays an important role in the regulation of host metabolism and protein translation in response to changing environmental conditions and the need for immunoregulation [12,13]. To investigate the activation of mTORC1 during EC infection with *Rickettsia* species, total protein lysates from *R. conorii*-infected ECs were subjected to Western blot analysis to investigate phosphorylation of a known mTORC1 substrate, namely p70 S6 kinase (p70 S6K). Cellular levels of p70 S6K phosphorylation in mock-infected ECs were very low to almost negligible as expected, but clearly abundant and significantly higher in response to *R. conorii* infection. The expression of total p70 S6K, however, remained relatively unaltered throughout the course of infection (Figure 1A). Quantitative analysis presented in Figure 1B clearly suggests an increase of about 3- to 4-fold in steady-state levels of phospho-p70 S6K on threonine 421/serine 424 in infected cells as early as 0.5 h and sustenance of this response up to 24 h post-infection in direct comparison to corresponding mock-infected controls (Figure 1A). These findings indicate activation of mTORC1 in response to in vitro *R. conorii* infection of human ECs.

**Figure 2:**
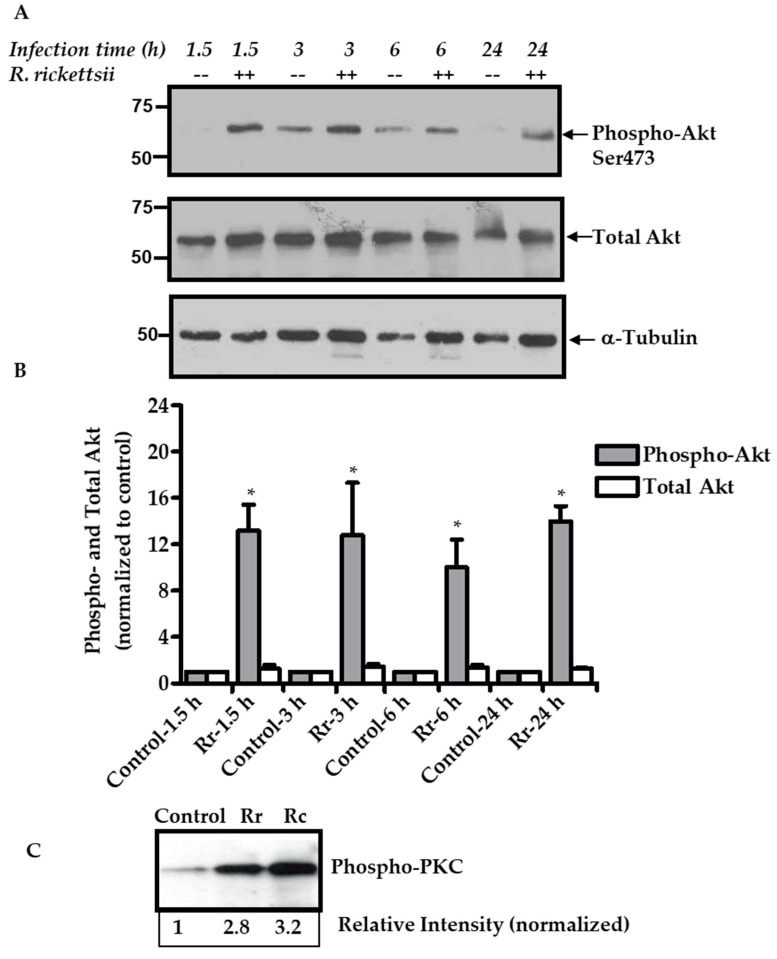
Mechanistic target of rapamycin complex 2 (mTORC2) activation in *R. rickettsii*-infected ECs. (**A**). Confluent monolayers of human microvascular endothelial cells (HMECs) were either mock infected (--) or infected with *R. rickettsii* (++) at MOI of 5 PFUs/cell for various times as indicated. Cellular protein lysates were prepared and processed for immunoblot analysis using antibodies against phospho- and total Akt. An antibody against α-tubulin was used to re-probe the blots as a loading control. Relative positions of the nearest molecular weight markers (kDa) from the protein ladder are indicated on the left flank of the gel. The results from a representative blot (*n* ≥ 3) are presented. (**B**). Quantitative densitometric analysis of phospho- and total Akt bands during *R. rickettsii* infection of ECs is shown as a function of time. The values are presented as the mean ± SEM from a minimum of three independent experiments. For comparison, basal levels in mock-infected cells (controls) were assigned a value of 1. The asterisk (*) indicates statistically significant changes (*p* ≤ 0.05) in *R. rickettsii*-infected cells compared with the baseline values in corresponding mock-infected controls. Data are representative of three independent experiments. A similar pattern of Akt phosphorylation was observed with *R. conorii*. (**C**). Phosphorylation of protein kinase C (PKC) after infection with *R. rickettsii* (Rr) or *R. conorii* (Rc) for 3 h in ECs.

To investigate the possibility of activation of mTORC2 in response to rickettsial infection of host ECs, we next determined the extent of phosphorylation of a known mTORC2 substrate, namely protein kinase B (Akt), at serine 473. Again, constitutive levels of phospho-Akt were very low or barely detectable in mock-infected ECs, but evidently much higher in infected cells (Figure 2). Quantitatively, phospho-Akt levels displayed an increase of about 10-fold or higher in infected cells as early as 1.5 h and remained elevated at the latest time of 24 h post-infection (Figure 2A). The expression levels of total Akt, however, did not demonstrate any significant changes during the infection. Detailed densitometric analysis for the quantitation of phospho- and total Akt in infected ECs is shown in Figure 2B. As a follow-up to confirm this finding, we further assessed the phosphorylation status of protein kinase C (PKC) as yet another substrate of mTORC2 to find a robust increase in cellular levels of phospho-PKC in response to both *R. rickettsii* and *R. conorii* infection (Figure 2C). This result is in concordance with our previous observations implicating selective involvement of specific isoforms of PKC in transcriptional responses (as measured by activation of NF-κB and induced expression and activity of tissue factor) of host ECs [14]. Taken together, these results demonstrate the activation of mTORC2 as well consequent to EC infection with spotted fever group rickettsiae.

**Figure 3:**
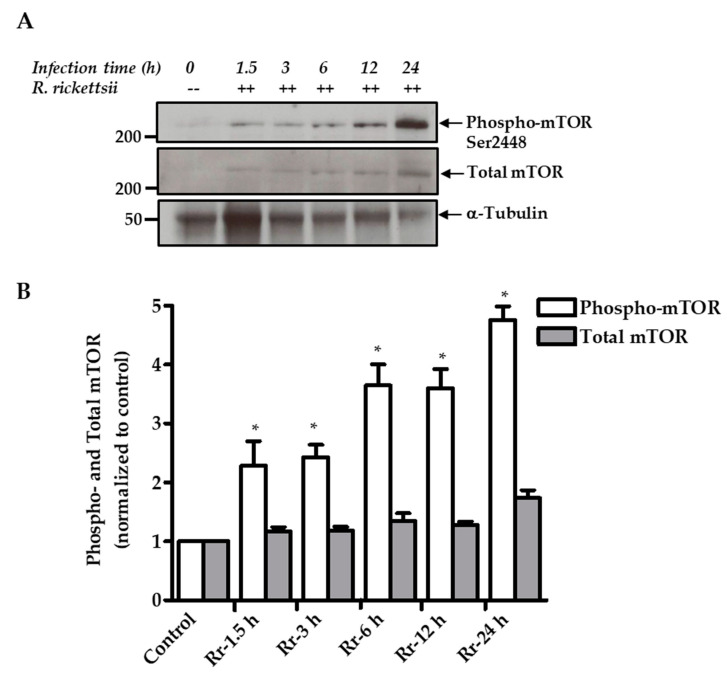
Mechanistic target of rapamycin (mTOR) phosphorylation in *R. rickettsii*-infected ECs. (**A**). Confluent HUVECs were either mock infected (--) or infected with *R. rickettsii* (++) at MOI of 5 PFUs/cell for various times as indicated. Total protein lysates from infected as well as corresponding mock-infected ECs were subjected to immunoblotting and probing with antibodies against phospho- and total mTOR. An antibody against α-tubulin was used to re-probe the blots after stripping to account for variations in sample loading on different lanes of the gel. Relative positions of nearest molecular weight marker (kDa) from the protein ladder are displayed on the left flank of the gel. Results from a representative blot (*n* ≥ 3) are shown. (**B**). Quantitative densitometry analysis of the phospho- and total mTOR during *R. rickettsii* infection of ECs as a function of time. The values are presented as the mean ± SEM of a minimum of three independent experiments. The asterisk (*) indicates statistically significant changes (*p* ≤ 0.05) in *R. rickettsii*-infected cells compared with the baseline values in the corresponding samples from uninfected ECs. Data are representative of three independent experiments.

Finally, we investigated the phosphorylation of mTOR itself on serine 2448 as a direct indicator of activation using an antibody capable of detecting phospho-mTOR. As shown, we noticed a pattern of time-dependent increase from 1.5 to 24 h (5.0 ± 0.5-fold) in the steady-state levels of mTOR phosphorylation in infected cells in direct comparison to mock-infected controls (Figure 3A). Again, the levels of total mTOR remain relatively unchanged through the course of infection. Further quantitative analysis from three independent experiments is depicted in Figure 3B. Thus, our findings clearly establish that in vitro infection of host ECs by pathogenic spotted fever rickettsiae induces the activation of both mTOR complexes designated as mTORC1 and mTORC2.

**Figure 4:**
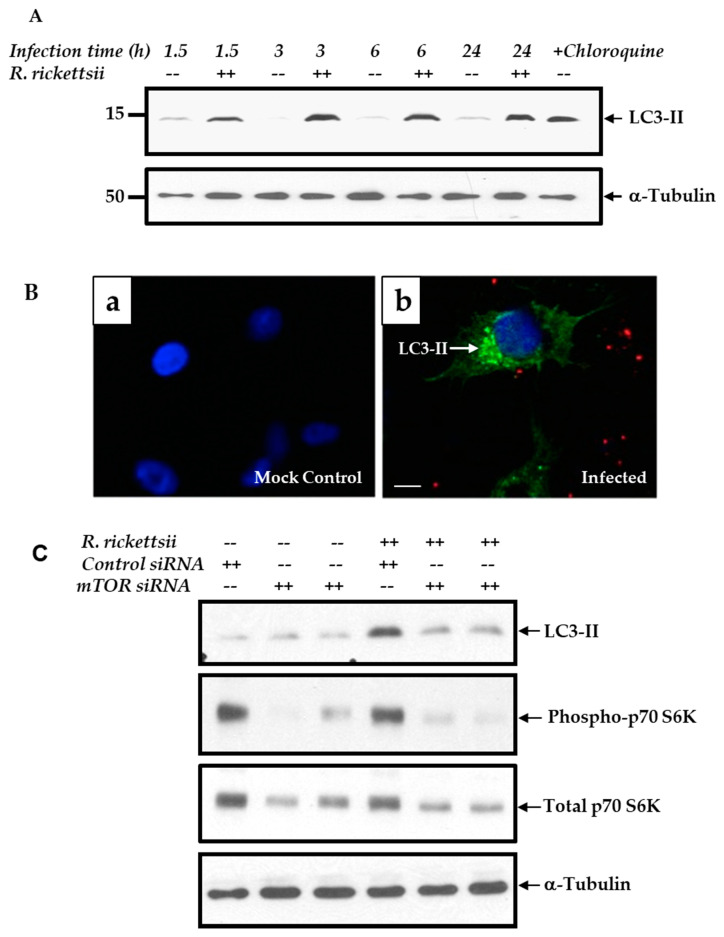
LC3-II lipidation in *R. rickettsii*-infected ECs. (**A**). Confluent ECs were either mock infected (--) or infected with *R. rickettsii* (++) for various times as indicated. Cell lysates were then prepared and subjected to immunoblot analysis with antibodies against LC3-II. An antibody to probe for α-tubulin was used as a loading control and relative positions of nearest molecular weight markers (kDa) from the protein ladder are indicated on the left flank of the gel. A representative blot (*n* ≥ 3) is presented. (**B**). ECs were plated on sterile plastic coverslips and either mock infected (**a**) or infected with *R. rickettsii* (**b**) for 6 h. Cells were washed, fixed, permeabilized, and stained for LC3-II using a rabbit polyclonal anti-LC3-II (Alexa Fluor 488, Green) and a guinea pig anti-*R. rickettsii* antibody (Alexa Fluor 568, Red). Cells were counterstained with DAPI to stain the nuclei (blue). Scale Bar = 5 μm. (**C**). ECs was transfected with 25 nM or 50 nM of mTOR siRNA along with 50 nM of control siRNA for 48 h prior to infection with rickettsiae for 24 h. Total protein lysates were used for performing Western blotting and resultant blots were probed with antibodies for the detection of LC3-II, phospho- and total p70 S6K, and α-tubulin. A representative blot (*n* ≥ 3) is shown.

Autophagy represents a fundamentally important regulatory mechanism resulting in the recycling of cellular constituents by lysosomes. Studies have shown involvement of a number of signal transduction pathways in the regulation of autophagy, including those converging at the mTOR, which is now well appreciated for a major role in the regulation of autophagy. Therefore, to investigate the potential link between mTOR and host cell autophagy in the context of rickettsial infection, we investigated the initial step in the activation of autophagic cascade by analyzing lipidation of microtubule-associated protein 1A/1B-light chain 3 (LC3-II). As shown in Figure 4A, LC3-II lipidation was enhanced in response to infection at all studied time points between 1.5 and 24 h post-infection, in comparison to simultaneously processed mock-infected controls. Treatment of cells with chloroquine was used as a positive control in these experiments. As the next step, mock- and *R. rickettsii*-infected ECs were stained for LC3-II to visualize the formation of autophagosomes. There was clear evidence for the formation of LC3-II puncta in infected cells (stained green and indicated by an arrow), while being absent or barely noticeable in the corresponding mock-infected controls (Figure 4B). The cell nuclei and rickettsiae in infected cells are clearly identifiable by blue and red fluorescence, respectively. Together, these findings yield further evidence suggesting that rickettsial infection triggers initial autophagic response in host ECs. To determine further whether induction of the autophagy cascade is governed by mTOR, we transfected ECs with either mTOR-specific or a control siRNA for 48 h prior to infection of cells with *R. rickettsii* and analysis of LC3-II lipidation. Interestingly, mTOR siRNA significantly downregulated the intensity of LC3-II lipidation in *R. rickettsii*-infected ECs as compared to mock-transfected controls (Figure 4B). To ensure the functionality of siRNA used, we also assessed cellular levels of both phospho- and total p70 S6 kinase to validate the attenuation of its phosphorylation in cells transfected with mTOR-specific, but not scrambled control siRNA (Figure 4C).

## 3. Discussion

Rickettsial invasion and replication within the cytoplasm of otherwise quiescent ECs result in their activation, culminating in the acquisition of a pro-adhesive, pro-coagulant, and pro-inflammatory phenotype [1,2,3]. Despite the ECs’ important roles as a prime contributor to host responses to rickettsial infections via recruitment of inflammatory phagocytes, endothelial involvement in self-protection via regulation of autophagy or xenophagy represents a neglected area of scientific inquiry. The mammalian/mechanistic target of rapamycin (mTOR) is now well established as a critical cellular regulator of autophagy and pro- vis-à-vis anti-inflammatory innate immune responses in eukaryotic host cells [5,9,15]. The present study illustrates activation of mTOR signaling—mediated by both Raptor-based C1 and Rictor-based C2 mTOR complexes—during infection of cultured human ECs with pathogenic rickettsiae. A number of other pathogenic bacteria, including *Listeria monocytogenes* [16], *Salmonella enterica* serovar Typhimurium (*S.* Typhimurium) [17], *Mycobacterium tuberculosis* [18], *Shigella flexneri* [19], and *Francisella tularensis* [20], exploit mTORC1 and/or mTORC2 to acquire entry into and establishing a proliferative niche in their target host cells. Thus, it is not surprising that pathogens interact with host mTOR pathway as a master regulator of cellular metabolism and its downstream signaling moieties to either finetune or subvert metabolic processes to acquire a supportive growth niche and replicative advantage within the host cell. As a serine/threonine protein kinase belonging to the PI3-kinase family, it remains plausible that mTOR signaling may have similar roles in rickettsial pathogenesis, since early activation of PI3-kinase has previously been implicated in the adhesion and entry of *R. conorii* into mammalian host cells [21]. Nonetheless, the findings of this study yield convincing first evidence for mTOR activation in target ECs infected in vitro with pathogenic spotted fever species *R. rickettsii* and *R. conorii*.

Ample evidence now supports a physiologically important link between mTOR and autophagy, which as a homeostatic mechanism facilitates recycling and/or degradation of damaged or dysfunctional organelles to ensure cell survival during nutrient deprivation or other stress conditions [22]. In the context of intracellular pathogens, however, induction of autophagy to clear the infection (xenophagy) and stimulate anti-inflammatory mechanisms constitutes an important host defense strategy to minimize the extent of host damage and immunopathology. Regulation of autophagy thus provides a crucial interface between pathogenesis and host defense, because intracellular pathogens in the family Rickettsiaceae not only consume host cell energy by ATP/ADP exchange, but also benefit from metabolic resources as has been documented for other obligatory intracellular bacteria belonging to *Anaplasma* and *Ehrlichia* species within the order Rickettsiales. Unlike free cytoplasmic rickettsiae, intracellular *Ehrlichia chaffeensis* remains confined to an early endosome-like membrane-bound compartment and delivers a type IV secretion system effector, *E. chaffeensis* translocated factor-1 (Etf1), to capture host-derived nutrients by co-opting autophagy for the sake of its proliferation and replication [23,24]. A similar strategy to acquire host nutrients for its growth in membrane-bound inclusion vacuoles has also been documented for *Anaplasma phagocytophilum* [24,25]. Our results showing robust lipidation of LC3-II as a widely accepted marker and accumulation of autophagosomes decorated by this protein in response to rickettsial infection of host ECs clearly demonstrate initiation of autophagic cascade, yet the success of rickettsiae as intracellular parasites capable of establishing intracellular infection and intercellular spread further suggests their ability to evade this intrinsic host defense mechanism to curb the infection.

mTOR is an established regulator of autophagy as a vitally crucial cellular mechanism of nutrient sensing and signaling. An important consideration in this regard is that while intracellular pathogens induce amino acid starvation to trigger autophagy and vice versa, activation of mTOR might function to dampen this response. Accordingly, our initial findings support the notion that rickettsiae likely exploit activation of mTOR during infection to interfere with the autophagic response of ECs. Published evidence implicates mTORC1 in the downregulation of autophagy induction and documents that its activity undergoes reactivation towards the end of autophagy by virtue of energy generation as a consequence of degradation of auto-lysosomal cargo, confirming a critical role for mTORC1 in the completion of this complex biological process [26]. Autophagy regulation by mTORC1 in mouse macrophages serves as an important defense mechanism against *M. tuberculosis,* *S.* Typhimurium, and *Pseudomonas aeruginosa*, resulting in the limitation of intracellular growth and replication of these pathogens [27,28,29,30]. Interestingly, signaling modules governed by Akt and mTOR in *S.* Typhimurium-infected mouse macrophages inhibit autophagy and promote bacterial survival [31]. Published evidence further suggests that *E. chaffeensis* also interferes with canonical autophagy through regulation of Wnt:PI3-kinase:mTOR signaling pathways via a repertoire of secreted tandem repeat proteins to ensure its survival and replication in the host cell by preventing its processing and destruction in phagolysosomes [32]. Similarly, viruses such as HIV-1 also induce attenuation of autophagy via mTOR pathway to evade host immune responses [33]. Although our findings suggesting potential involvement of mTORC1 in the inhibition of autophagy during *Rickettsia* infection of ECs are in agreement with those for other pathogens, potential roles of mTORC2 in the control of autophagy/xenophagy during infection warrant further investigation.

As a versatile signaling moiety, mTOR also serves as a critically important determinant of inflammation by virtue of regulation of NF-κB- and STAT-mediated host responses. Previous work from our laboratory has unequivocally established activation of both NF-κB and STAT-3 or STAT-1 as well as expression and secretion of cytokines (IL-1 and interferon-β) and chemokines (IL-8 and MCP-1) by ECs infected with *R. rickettsii* and *R. conorii* [1,2,3,10,11,14]. Increased cytokine production in response to invading pathogens may serve to sustain the inflammatory immune response by maintaining the recruitment of infiltrating macrophages and/or neutrophils to the foci of infection. Evidence also suggests, however, that bacteria such as *Staphylococcus aureus* and *Listeria monocytogenes* activate mTOR signaling to induce the production of the anti-inflammatory cytokine IL-10, promoting their survival in the host [9,34]. Because our recent preliminary findings indicate a trend for increased mRNA expression of IL-10 by ECs infected with *R. conorii* (based on steady-state transcript levels below the limit of detection with undetermined Ct values in mock-infected controls versus distinguishable average Ct values of 35 and 36 in cells infected for 3 and 24 h, respectively), it is reasonable to rationalize that mTOR may also contribute to the balance of pro- versus anti-inflammatory mediators during rickettsial infection of host cells via regulation of NF-κB, STAT, and possibly other signaling modules. Our currently ongoing studies focused on interference with downstream signaling targeting mTORC1 and/or mTORC2 at a molecular level and global or complex-specific inhibition using pharmacological inhibitors such as rapamycin or torins should further advance our understanding of important determinants of host responses to rickettsial pathogens.

## 4. Materials and Methods

### 4.1. Cell Culture and Infection

Primary cultures of human umbilical vein endothelial cells (HUVECs) were established using procedures established in our laboratory [10]. The cells were cultured in McCoy’s 5A medium (Thermo Fisher Scientific, Waltham, MA, USA) supplemented with fetal bovine serum (FBS) (20% *v*/*v*, Aleken Biologicals, Nash, TX, USA), endothelial cell growth supplement (ECGS) (50 μg/mL, Thermo Fisher), heparin (100 μg/mL, MilliporeSigma, St. Louis, MO, USA), and L-glutamine (10 mM, Thermo Fisher). HMECs, represented by an immortalized cell line HMEC-1 derived from human dermal microvascular endothelial cells, were grown in MCDB-131 medium (Caisson Labs, Smithfield, UT, USA) containing FBS (10% *v*/*v*), epidermal growth factor (10 ng/mL, Thermo Fisher), hydrocortisone (1 μg/mL, MilliporeSigma), and L-glutamine (10 mM, Thermo Fisher) as described previously [35]. *R. rickettsii* (Strain Sheila Smith) and *R. conorii* (Malish7) were propagated in Vero cells, purified by centrifugation after gentle cell lysis using glass beads, and kept frozen at −80 °C as aliquoted stocks of ~0.5 mL to avoid the loss of viability due to repeated freeze–thaw cycles. Infectivity titers of purified stocks were estimated by citrate synthase (*gltA*)-based quantitative PCR and plaque formation assay on Vero cells [11,35]. ECs at about 80–90% confluence were infected with either *R. rickettsii* or *R. conorii* (MOI = 5) according to published protocols and procedures [36]. At different times post-infection, total cell lysates were prepared by thorough washing of cells with cold phosphate-buffered saline (PBS), gentle scraping in a lysis buffer containing SDS (0.2% *w*/*v*) and inhibitor cocktails for protease and phosphatase activities, and mild sonication on ice. The viability of mock controls and infected ECs in all experiments was routinely ascertained microscopically. In some experiments, mTOR-specific (25 or 50 nM) or control siRNA (50 nM) obtained from Cell Signaling Technology, Danvers, MA, USA, were transfected into ECs using Lipofectamine 2000 (Thermo Fisher Scientific) according to the manufacturer’s instructions for 48 h prior to infection followed by the preparation of cell lysates as described above.

### 4.2. Western Blotting

Equal volumes of total protein lysates were separated on a denaturing polyacrylamide gel, which were then processed for wet transfer onto a nitrocellulose membrane followed by probing of the blots with antibodies to detect phospho-p70 S6 kinase (Thr421/Ser424), phospho-Akt (Ser473), phospho-mTOR (Ser2448), phospho-PKC, total p70 S6 kinase, total Akt, total mTOR, and LC3-II (Cell Signaling Technology) and a compatible horseradish peroxidase (HRP)-conjugated secondary antibody for enhanced chemiluminescence-based detection. To normalize for variations in the protein loading on individual lanes, the blots were stripped and re-probed with an antibody against α-tubulin (Accurate Chemical and Scientific Corporation, Westbury, NY, USA).

### 4.3. Immunofluorescent Staining

ECs were seeded on glass coverslips and infected with *R. rickettsii* as described above. At 6h post-infection, cells were washed with PBS, fixed with 3.7% formaldehyde, permeabilized with 0.2% Triton X-100 for 20 min, and incubated with Image-iTTM FX signal enhancer (Thermo Fisher) to block any background staining. The cells were then incubated with a rabbit polyclonal antibody against LC3-II (Cell Signaling) followed by an Alexa Fluor 488 secondary antibody. Cells were also stained with an anti-*R. rickettsii* antibody followed by a compatible Alexa Fluor 568 secondary antibody. Nuclei were stained with 4′,6-diamidino-2-phenylindole (DAPI). The staining was observed using a FluoView^®^ FV10i Olympus confocal microscope and software (Center Valley, PA, USA) and images were captured using a camera system connected to the microscope.

### 4.4. Densitometry and Statistical Analysis

The autoradiograms for Western blots were scanned at a resolution of 300 dpi and band intensity was calculated using Image Studio Lite software (version 5.2, Lincoln, NE, USA) and normalized to α-tubulin. Basal levels of expression in mock-infected controls was assigned a value of 1 for comparison with experimental conditions. All experiments were performed at least three times with a minimum of two technical duplicates to calculate the results as the mean ± standard error of the mean (SEM). Statistical significance between groups was evaluated by Student’s *t*-test and/or one-way analysis of variance (ANOVA) with Dunnett’s post-test using GraphPad Prism 4.00 (GraphPad software, San Diego, CA, USA). The threshold for significant changes was set at a *p*-value of ≤ 0.05.
